# Identification of intrinsic genes across general hypertension, hypertension with left ventricular remodeling, and uncontrolled hypertension

**DOI:** 10.3389/fcvm.2022.992284

**Published:** 2022-10-06

**Authors:** Chun-yang Yu, Yang Gu, Yi-cheng Jiang, Xi-wen Zhang

**Affiliations:** Department of Cardiology, The Affiliated Huaian No.1 People's Hospital of Nanjing Medical University, Huai'an City, China

**Keywords:** intrinsic genes, hypertension, left ventricular remodeling, uncontrolled hypertension, bioinformatics strategies

## Abstract

The purpose of the present article is to identify intrinsic genes across general hypertension (HT), hypertension with left ventricular remodeling (HT-LVR), and uncontrolled hypertension (UN-HT). In total, four microarray datasets (GSE24752, GSE75360, GSE74144, and GSE71994) were downloaded from the GEO database and were used to identify differentially expressed genes (DEGs), respectively. Furthermore, gene set enrichment analysis (GSEA) was utilized to screen for significantly enriched biological pathways across the four datasets above, respectively. Furthermore, weighted gene co-expression network analysis (WGCNA) and functional enrichment analysis were applied to screen out gene modules of interest and potential biological functions, respectively. Finally, a Metascape-based multiple gene list meta-analysis was used to investigate intrinsic genes at different stages of the progression of hypertension. A total of 75 DEGs (63 upregulated genes and 12 downregulated genes, GSE24752) and 23 DEGs (2 upregulated genes and 21 downregulated genes, GSE74144) were identified. However, there were few DEGs identified in GSE75360, GSE71994, and part of the GSE74144 datasets. GSEA and functional enrichment of gene module of interest have indicated that “Heme metabolism,” “TNF alpha/NFkB,” and “interferon alpha response signaling,” and MYC target v1/v2 were enriched significantly in different stages of hypertension progression. Significantly, findings from the multiple gene list meta-analysis suggested that *FBXW4* and other 13 genes were unique to the hypertension group, and *TRIM11* and other 40 genes were mainly involved in hypertension with the left ventricular remodeling group, while the other 18 genes including *F13A1* significantly enriched in uncontrolled hypertension. Collectively, the precise switch of the “immune-metabolic-inflammatory” loop pathway was the most significant hallmark across different stages of hypertension, thereby providing a potential therapeutic target for uncontrolled hypertension treatment.

## Introduction

Hypertension (HT) has been the most common public-health cardiovascular disease with a high incidence and a range of changeable and complex complications worldwide (1). Moreover, HT is a complex systemic disease, which not only endangers the cardiovascular system, but also damages other organs and systems (2). Whereas, current treatment methods for hypertension, which greatly depend on long-acting antihypertensive drugs given one time daily, are discouraging (3).

Advances in high-throughput screening technology and novel bioinformatics algorithms have facilitated the systematic detection of the research objects (4). Furthermore, a classic example of high throughput technology applied to hypertension research was a genetic analysis that identified 535 new loci associated with hypertension in over 1 million people (5).

Cardiac remodeling especially left ventricular myocardial remodeling is considered one of the hallmarks of hypertensive heart disease progression(6). In addition, Ehlers et al. (7) suggested the activation of AT1R triggers cardiovascular remodeling in subjects with hypertension. An expressive number of patients continue with uncontrolled hypertension (UN-HT), despite the availability of many antihypertensive drugs and various guidelines on the management (8, 9). A multicenter study (10) suggested that many variants of these genes were present in uncontrolled hypertension.

This is the first data-mining study to systematically identify peripheral blood transcriptome characteristics of hypertension at different stages, such as hypertension, hypertension with left ventricular remodeling (HT-LVR), and uncontrolled hypertension by means of bioinformatics methods. Liew et al. (11) proposed that expression levels of most genes between bulk tissues and peripheral blood have a good positive linear correlation, demonstrating that peripheral blood is an ideal surrogate tissue to discover genes and pathways. Compared with tissue samples, blood samples have great advantages in early clinical disease diagnosis.

In terms of research methods, we prefer to perform a more robust gene set enrichment analysis (GSEA) rather than the traditional enrichment analysis based on hypergeometric distribution to find transcript functions. Our core step was to detect modules of interest through the weighted gene co-expression network analysis (WGCNA) and adopted a multi-gene list meta-analysis method to identify intrinsic genes and their interaction at different stages of the progression of hypertension.

Collectively, our study suggested that the signal conversion of the immune-metabolic-inflammatory loop pathway was the most significant hallmark at different stages of hypertension, and identify potential targets for uncontrolled hypertension treatment.

## Materials and methods

### Candidate dataset collection and preprocessing

We collected hypertension-related transcriptomic datasets with complete expression profiles and experimental design information from the Gene Expression Omnibus (12) (GEO) database in the present study by using the keyword “hypertension.”

The datasets GSE24752, GSE74144, and GSE71994 were downloaded using the R package (13) “GEOquery” (v2.60.0). For the GSE75360 dataset, the non-normalized expression matrix was downloaded from the GEO repository.

The GSE24752 dataset (14) contains 3 hypertension and 3 normal human gene expression data (GPL570, peripheral blood cells, Affymetrix Human Genome U133 Plus 2.0 Array, Affymetrix Inc., Santa Clara, CA, USA).

The GSE75360 dataset (15) contains 10 hypertension and 11 normal human gene expression data (Illumina HumanHT-12 v.4.0 Expression BeadChip, Illumina Inc., San Diego, CA, USA, GPL10558, peripheral blood mononuclear cells).

The GSE74144 dataset includes 14 patients with hypertension and left ventricular hypertrophy, 14 patients with hypertension and normal left ventricular size, and 8 control individuals' gene expression data [Agilent-026652 Whole Human Genome Microarray 4x44K v2 (Probe Name version), Agilent Technologies, Palo Alto, CA, USA, GPL13497, white blood cells].

The GSE71994 dataset is composed of 20 controlled and uncontrolled hypertensive patients' gene expression data (Affymetrix Human Gene 1.0 ST Array (transcript (gene) version), Affymetrix Inc., Santa Clara, CA, USA, PMID: 27779208, GPL6244, peripheral blood mononuclear cells).

The R packages “hgu133plus2.db” (v3.13.0), “illuminaHumanv4.db” (v1.26.0), “hsAgilentDesign026652.db” (v3.2.3), and “hugene10sttranscriptcluster.db” (v 8.8.0) were used to convert gene probes to gene symbols according to the microarray platform, respectively, and then all gene names were remapped to official gene symbols according to the multi-symbol checker tools (https://www.genenames.org/tools/multi-symbol-checker/). On the basis of the experimental design, only GSE75360, GSE74144, and GSE71994 were selected for the subsequent analysis of weighted gene co-expression network analysis (WGNCA).

Quartile normalization was applied to the gene expression matrix using “normalizeBetweenArrays” of the R packages (16) “limma” (v3.48.3) and a Log2 transformation was conducted for subsequent data analysis. For genes with multiple probes, the median value from all expressed probes was used. When one probe was matched to multiple genes, it was deleted. Specifically, genes with a zero expression were excluded from all samples.

### Screening of differentially expressed genes (DEGs) and setting an optimal threshold

We used the R package “limma” (v3.48.3) to identify differentially expressed genes (DEGs) between the control group and the experimental group from the GSE24752, GSE75360, GSE74144, and GSE71994 datasets.

Since alterations of RNA levels are usually lower in peripheral-blood samples and peripheral blood mononuclear cells than in other tissues (17), we performed R package “RVA” (v0.0.4) to visualize the number of DEGs under different thresholds. In addition, an absolute 2-fold change with a *p-*value of < 0.05 was considered DEGs.

### Gene set enrichment analysis (GSEA)

A Gene set enrichment analysis (GSEA) was performed using the R package (18) “fgsea” (Version 1.18.0) with parameters: minSize = 10, maxSize = 2,000, nperm = 10,000 using MSigDB H: hallmark gene sets (50 gene sets available, V7.4). The gene-pathway data file is available for download on the publicly available MSigDB website (19): http://www.gsea-msigdb.org/gsea/msigdb/index.jsp. Gene sets with a false discovery rate (FDR) value of < 0.05 and a normalized enrichment score (NES) of > 1 & −1 were considered significantly enriched.

### Identification of co-expression modules of interest using WGCNA

A weighted gene co-expression network analysis (WGCNA) was designed to identify interesting trait-related, such as dataset preprocessing, soft powers selection, constructing adjacency matrix and topological overlap matrix (TOM) construction, hierarchical clustering, and cluster partition.

The GSE24752 dataset failed to perform weighted gene co-expression network analysis (WGCNA) owing to too few samples for both phenotypes. The gene co-expression networks of the GSE75360, GSE74144, and GSE71994 datasets were constructed by the R package (20) “WGCNA” (v1.70-3).

First, the “pickSoftThreshold” function was used to select optimal soft powers to establish a scale-free network. Next, we calculated an adjacency matrix and corresponding topological overlap matrix (TOM), and hierarchical clustering to find genes with similar expressions in one co-expression module based on the one-step-WGCNA method in the “blockwiseModules” function. Finally, we investigated the module-trait relations between modules and external traits to find functional modules in this co-expression network by the “labeledHeatmap” function. Collectively, certain modules with the highest correlation coefficient and significant *p*-value were considered the candidate module that is closely correlated with the interested traits, and we used this module for our subsequent analysis.

### Functional enrichment analysis of gene modules of interest

The functional enrichment analysis based on the “Metascape” (Database Last Update Date: 2021-08-01) platform (21), which included Gene Ontology (GO)/Kyoto Encyclopedia of Genes and Genomes (KEGG) and transcript factors enrichment analysis, was performed using the genes involved in the modules that are closely correlated with the interested traits in WGCNA. Default settings with genes with an FDR of < 5% were considered significant terms.

### Multi-gene list meta-analysis

In general, Venn diagrams methods were used routinely to identify hits that were common or unique to certain gene lists. However, Chanda SK et al. proposed a multi-gene set analysis method that can better reflect the biological significance using the “Metascape” (Database Last Update Date: 2021-08-01) platform (21).

Therefore, we identified intrinsic gene sets across general hypertension, hypertension with left ventricular remodeling, and uncontrolled hypertension by means of the “Multi-gene list meta-analysis” methods. The specific steps include three steps, that is, protein–protein interaction (PPI) network construction, functional enrichment analysis, and hub genes selection. The protein–protein interaction (PPI) network of gene modules of interest was established through the Metascape platform. The most significant gene module from the PPI network was visualized and shown using the Molecular Complex Detection (MCODE) method. Cytoscape software (22) (http://www.cytoscape.org/, v3.8.2) was applied to construct a visual network of molecular interactions.

### Statistical analysis

Most of the statistical analyses were conducted using R software version 4.1.1. All the *p-*values and adjusted *p-*values were for a two-sided test and considered statistically significant when *p* was < 0.05.

## Results

### Identification of HT-DEGs, HT-LVR -DEGs, and UN-HT

**Figure 1:**
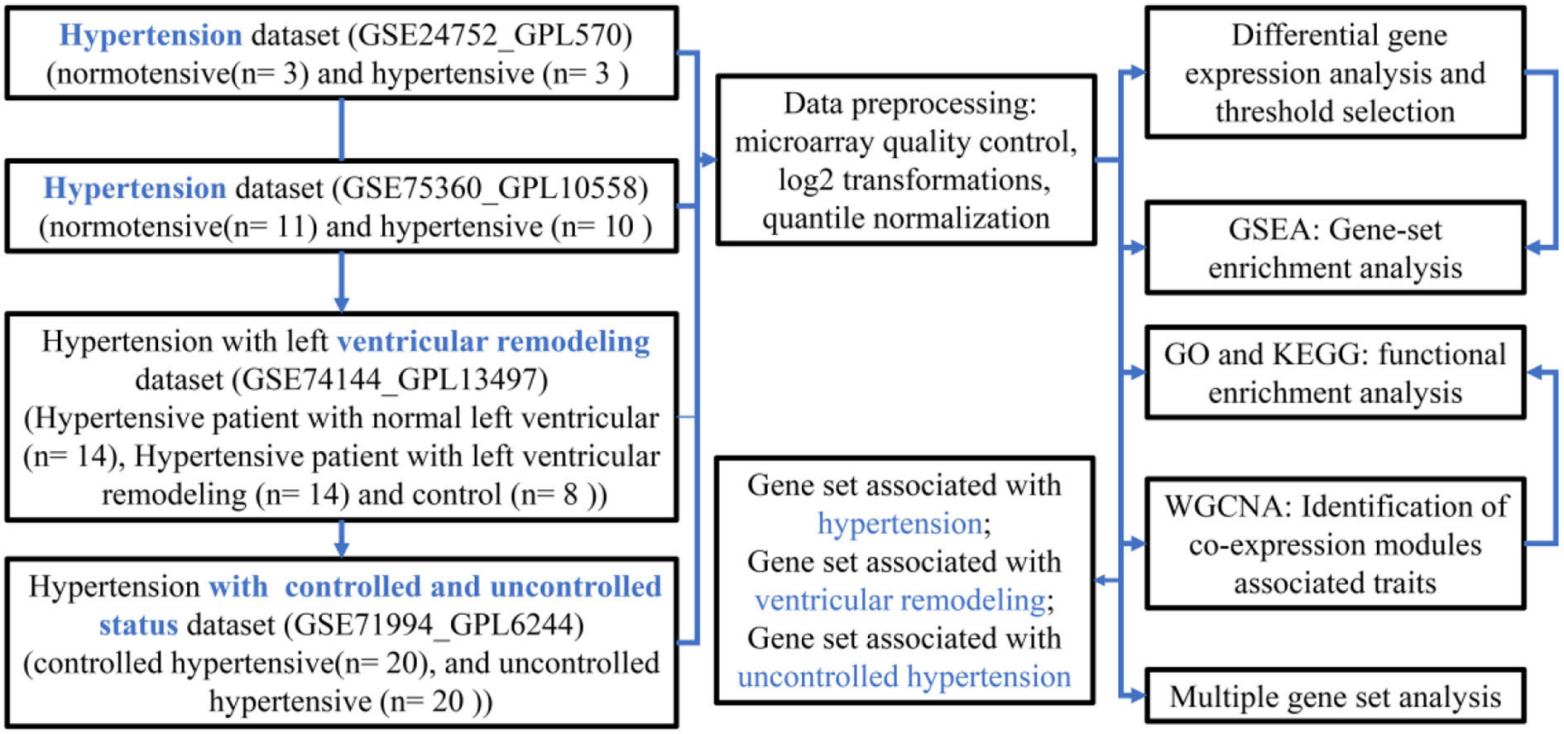
A flowchart diagram of hypertension-related transcriptional dataset selection, pre-processing, and analysis for our study.

**Figure 2:**
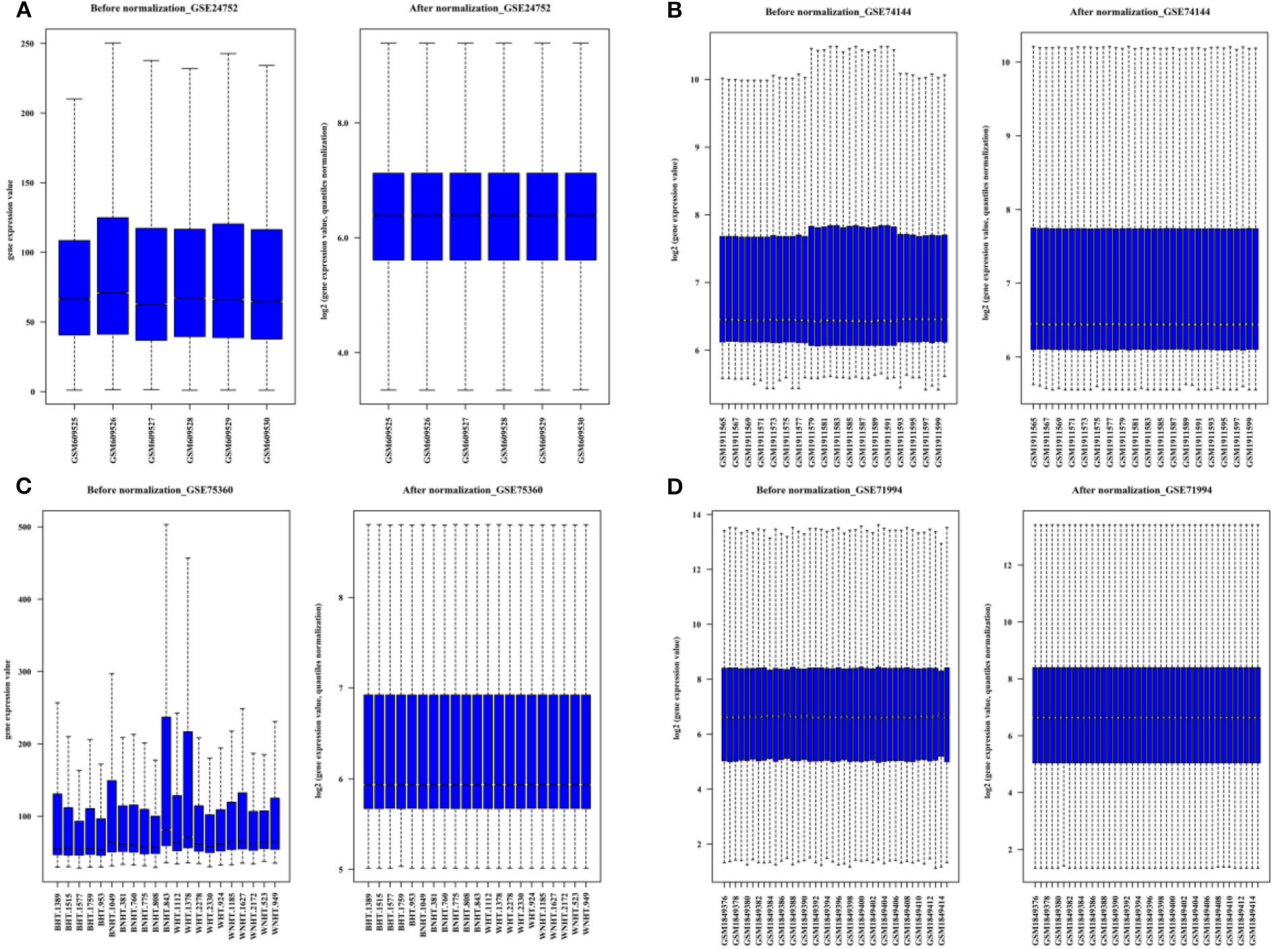
The boxplots for the microarray dataset prior and subsequent to normalization. **(A)** GSE 24752: Normotensive (*n* = 3) and hypertensive (*n* = 3). **(B)** GSE75360: Normotensive (*n* = 11) and hypertensive (*n* = 10). **(C)** GSE74144: Hypertensive patient with normal left ventricular (*n* = 14), hypertensive patient with left ventricular remodeling (*n* = 14) and control (n = 8). **(D)** GSE71994: Controlled hypertensive (*n* = 20), and uncontrolled hypertensive (*n* = 20).

The flow diagram of the current study is presented in Figure 1, which mainly includes the transcriptomic dataset download and preprocessing (Figure 2), function enrichment analysis, and WGCNA.

According to the microarray groups design, the comparison matrix for differential gene expression analysis was set as follows: GSE24752: hypertensive (*n* = 3) vs. normotensive (*n* = 3); GSE75360: hypertensive (*n* = 10) vs. normotensive (*n* = 11); GSE71994: uncontrolled hypertensive (*n* = 20) vs. controlled hypertensive (*n* = 20); GSE74144: hypertensive patient with normal left ventricular (*n* = 14) vs. normal control (*n* = 8); GSE74144: hypertensive patient with left ventricular remodeling (*n* = 14) vs. normal control (*n* = 8); and GSE74144: hypertensive patient with left ventricular remodeling (*n* = 14) vs. hypertensive patient with normal left ventricular (*n* = 14).

**Figure 3:**
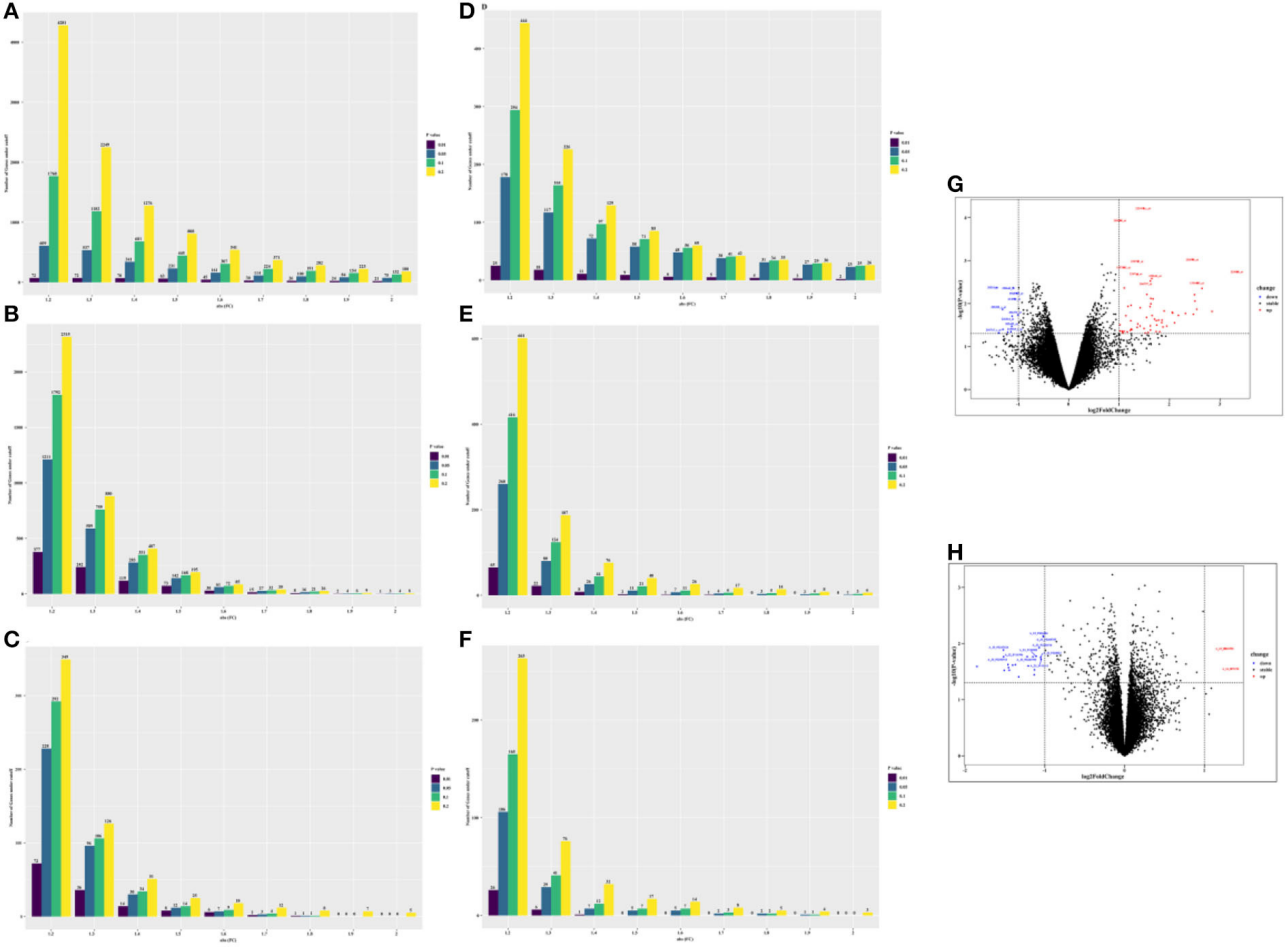
The number of differentially expressed genes (DEGs) under different thresholds [*p*-value and fold change (FC)] and corresponding volcano plot [fold change (FC) ≥ 2 and ≤ −2, and *p*-value of < 0.05 were used]. **(A)** GSE 24752: hypertensive (*n* = 3) vs. normotensive (*n* = 3). **(B)** GSE75360: hypertensive (*n* = 10) vs. normotensive (*n* = 11). **(C)** GSE71994: uncontrolled hypertensive (*n* = 20) vs. controlled hypertensive (*n* = 20). **(D)** GSE74144: hypertensive patient with normal left ventricular (*n* = 14) vs. normal control (*n* = 8). **(E)** GSE74144: hypertensive patient with left ventricular remodeling (*n* = 14) vs. normal control (*n* = 8). **(F)** GSE74144: hypertensive patient with left ventricular remodeling (*n* = 14) vs. hypertensive patient with normal left ventricular (*n* = 14). **(G)** Volcano plot for GSE24752: hypertensive (*n* = 3) vs. normotensive (*n* = 3). **(H)** Volcano plot for GSE74144: hypertensive patient with normal left ventricular (*n* = 14) vs. normal control (*n* = 8).

In total, we obtained 75 differentially expressed probes (DEGs, 63 upregulated genes, and 12 downregulated genes) in peripheral blood cell samples from the GSE24752 dataset (Figures 3A,G), which was consistent with Wei et al. (23). Furthermore, we identified 23 differentially expressed probes (DEGs, 2 upregulated genes, and 21 downregulated genes) in peripheral blood cell samples from the GSE74144 dataset (hypertensive patient with normal left ventricular vs. normal control), which was exhibited in Figures 3D,H. Interestingly, we detected few differentially expressed genes in the GSE75360, GSE71994, and part of the GSE74144 datasets based on an absolute 2-fold change with a *p*-value of < 0.05 (Figures 3B,C,E,F), which was contrary to that of Pang et al. (24) who found that 842 DEGs were identified in the GSE71994 dataset (including 629 upregulated genes and 213 downregulated genes), while 28,232 DEGs were identified in the GSE74144 dataset. To account for that contradiction, we continued searching for different publications. Li et al. (25) suggested lowering the threshold to increase the number of differentially expressed or adopting the weighted gene co-expression network analysis (WGCNA) algorithm. Therefore, we respectively performed a more robust gene set enrichment analysis (GSEA) and gene co-expression network analysis (WGCNA) to explore gene modules of interest. The full gene list is shown in Supplementary Table 1.

### Multiple signaling pathways were significantly enriched in different stages of hypertension progression

By using adaptive Monte Carlo sampling, FGSEA (18) was faster at enrichment analysis and more accurate at estimating low GSEA *p*-values than conventional GSEA (26).

**Figure 4:**
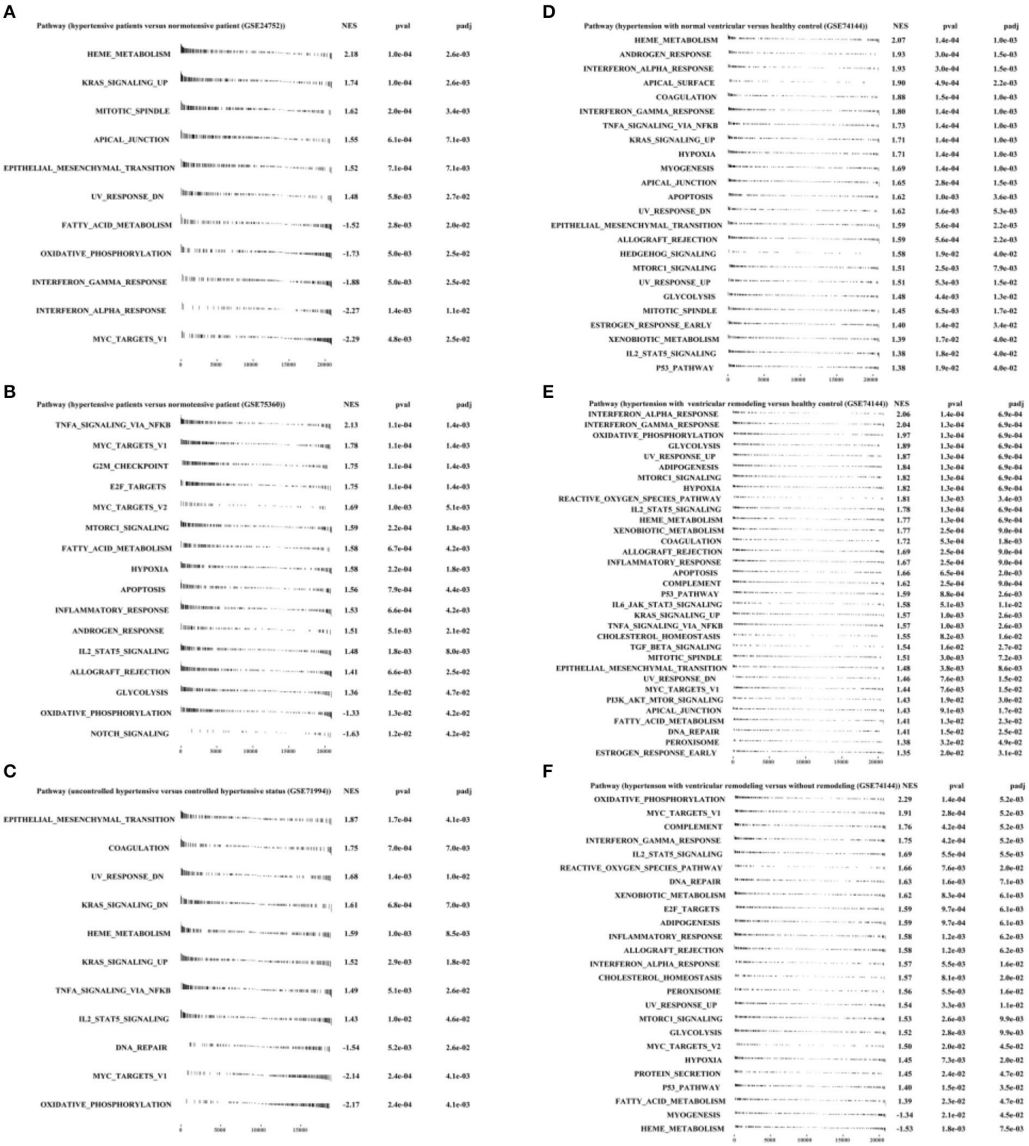
Fast pre-ranked gene set enrichment analysis (GSEA) based on h.all.v7.4.symbols.gmt (http://www.gsea-msigdb.org/gsea/msigdb/collections.jsp#H) by “fgsea” R packages. **(A)** GSE24752: hypertensive (*n* = 3) vs. normotensive (*n* = 3). **(B)** GSE75360: hypertensive (*n* = 10) vs. normotensive (*n* = 11). **(C)** GSE71994: uncontrolled hypertensive (*n* = 20) vs. controlled hypertensive (*n* = 20). **(D)** GSE74144: hypertensive patient with normal left ventricular (*n* = 14) vs. normal control (*n* = 8). **(E)** GSE74144: hypertensive patient with left ventricular remodeling (*n* = 14) vs. normal control (*n* = 8). **(F)** GSE74144: hypertensive patient with left ventricular remodeling (*n* = 14) vs. hypertensive patient with normal left ventricular (*n* = 14).

Figures 4A,D showed that HEME_ METABOLISM, KRAS_ SIGNALING_ UP, MITOTIC_ SPINDLE, and so on were significantly enriched in the hypertensive group, while MYC_ TARGETS_ V1 and OXIDATIVE_ PHOSPHORYLATION were enriched in the normotensive group, contrary to the result in Figure 4B. Due to the small sample size of the GSE24752 dataset and previous research findings, we are more willing to believe the results of Figures 4B,D.

Furthermore, Figure 4C exhibited that genes derived from uncontrolled hypertension were closely correlated with EPITHELIAL-MESENCHYMAL_ TRANSITION and COAGULATION, UV_ RESPONSE_ DN and DNA_ REPAIR, and TNFA_ SIGNALING_ VIA_ NFKB and IL2_ STAT5_ SIGNALING.

In addition, Figures 4E,F illustrated that INTERFERON_ ALPHA_ RESPONSE, INTERFERON_ GAMMA_ RESPONSE, GLYSCOLYSIS, and DNA REPAIR were significantly enriched in hypertension with left ventricular remodeling group.

### Selection of gene modules of interest at different stages of hypertension and functional analysis

Impressed by the findings of Li et al. (25), we focused on the gene modules at different stages of hypertension by means of WGCNA methods.

**Figure 5:**
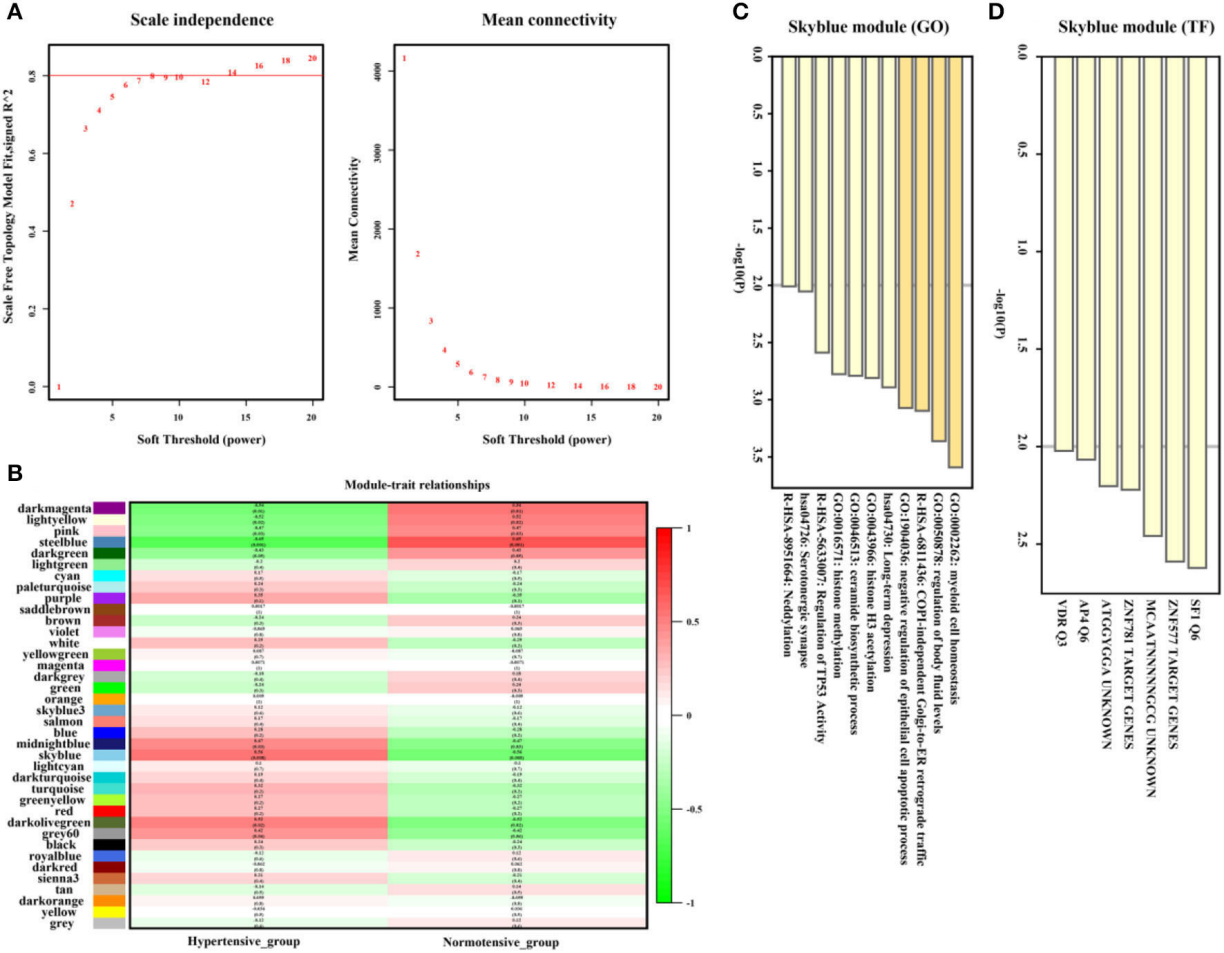
Hypertension-related gene module selection and functional enrichment analysis (GSE75360). **(A)** Robust threshold value to the selection. **(B)** A heatmap displaying the associated with hypertension trait and neighboring genes modules. **(C)** The Gene Ontology (GO) and Kyoto Encyclopedia of Genes and Genomes (KEGG) enrichment analysis for optimal hypertension-related gene module. **(D)** Transcript factors enrichment analysis for an optimal hypertension-related gene module.

As shown in Figures 5A,B, the SkyBlue gene module exhibited strong positive correlation with hypertension (*r* = 0.56, *p* = 0.008, soft threshold beta = 8). Genes from the SkyBlue gene module (Figure 5C) were significantly enriched in myeloid cell homeostasis, regulation of body fluid levels, COPI-independence Golgi-to-ER retrograde traffic, and so on, and which were widely monitored by SF1 and ZNF577 transcription factors (Figure 5D). The full list of genes is shown in Supplementary Table 2.

**Figure 6:**
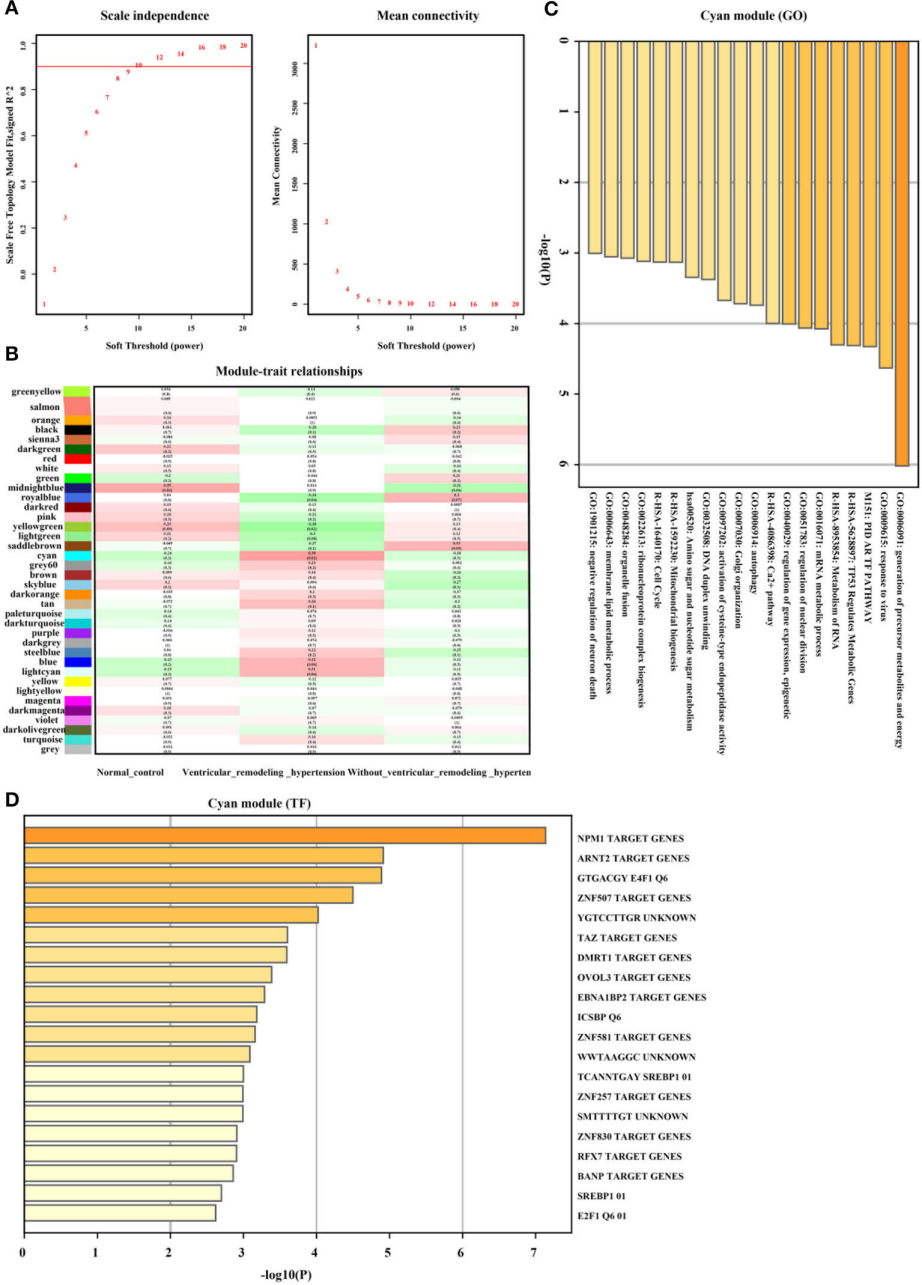
Hypertension with left ventricular remodeling related gene module selection and functional enrichment analysis (GSE74144). **(A)** Robust threshold value to the selection. **(B)** A heatmap displaying the association with hypertension with left ventricular remodeling trait and neighboring genes modules. **(C)** The GO and KEGG enrichment analysis for optimal hypertension with left ventricular remodeling-related gene module. **(D)** Transcript factors enrichment analysis for optimal hypertension with left ventricular remodeling-related gene module.

Furthermore, as illustrated in Figures 6A,B the Cyan gene module showed a strong positive correlation between hypertension and the left ventricular remodeling group (*r* = 0.39, *p* = 0.02, soft threshold beta = 10). Genes from the Cyan gene module (Figure 6C) were significantly enriched in the generation of precursor metabolites and energy, TP53 regulated metabolic genes, Ca^2+^ pathway, and so on, which were widely ruled by MPM1, ARNT2, and ZNF507 transcription factors (Figure 6D). The full list of genes is shown in Supplementary Table 3.

**Figure 7:**
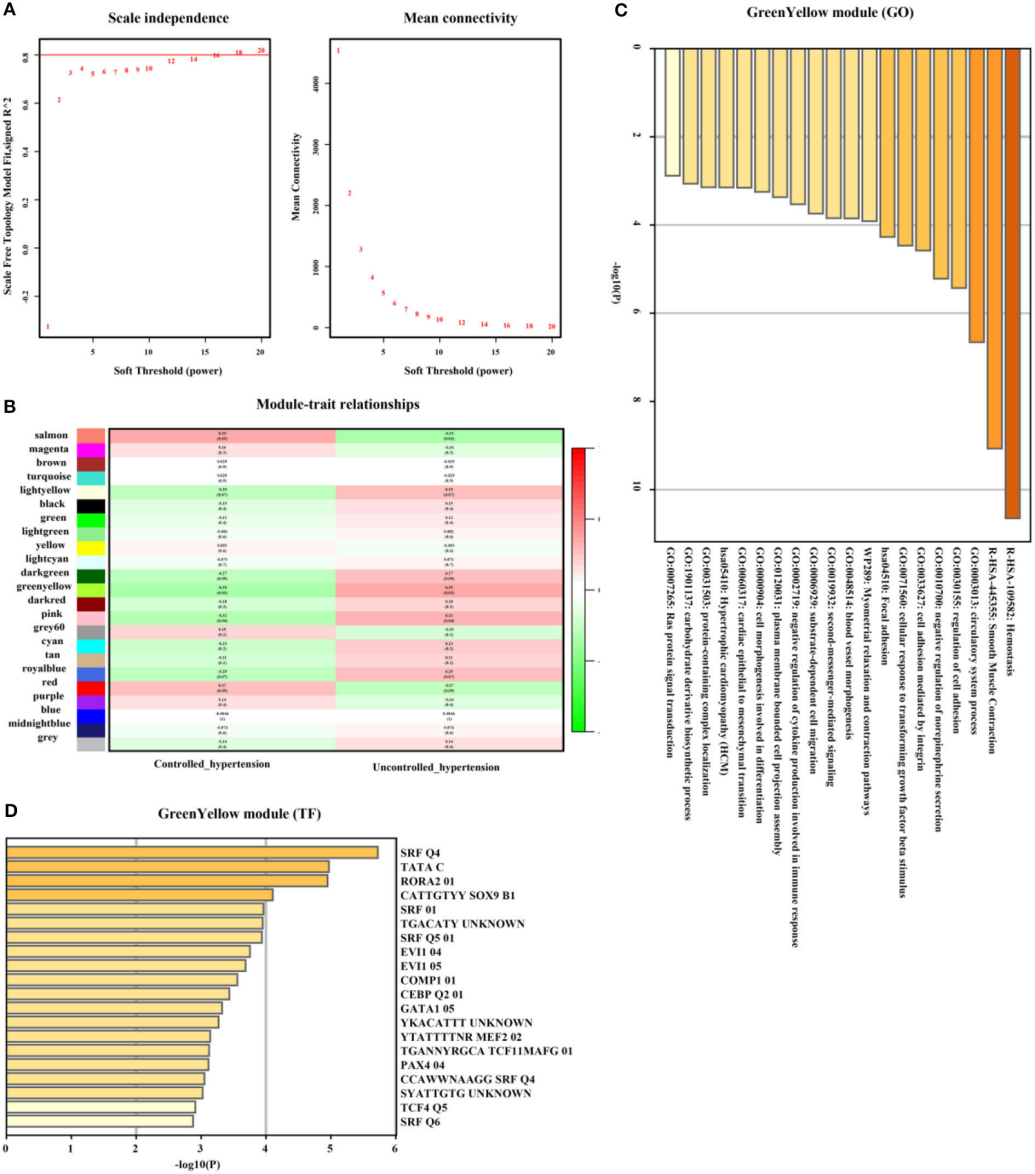
Uncontrolled hypertension-related gene module selection and functional enrichment analysis (GSE71994). **(A)** Robust threshold value to the selection. **(B)** A heatmap displaying the associated with uncontrolled hypertension trait and neighboring genes modules. **(C)** The GO and KEGG enrichment analysis for an optimal uncontrolled hypertension-related gene module. **(D)** Transcript factors enrichment analysis for an optimal uncontrolled hypertension-related gene module.

In addition, genes from GreenYellow, which were found to have the highest association with uncontrolled hypertension (*r* = 0.35, *p* = 0.03, soft threshold beta = 12, Figures 7A,B) were involved in hemostasis, smooth muscle contraction, regulation of cell adhesion, Ras protein signal transduction, and so on, which were broadly regulated by SRF and TCF4. Genes from the Magenta module (Figure 7C) were involved in lymphocyte activation and lymphocyte migration and were broadly regulated by nuclear factor-κB 1 (NFKB1) and signal transducer and activator of transcription 4 (STAT4) (Figure 7D). The full list of genes is shown in Supplementary Table 4.

### Identification of intrinsic gene set across HT, HT-LVR, and UN-HT

We performed a multi-gene-list meta-analysis across HT, HT-LVR, and UN-HT by means of the Metascape platform (21).

**Figure 8:**
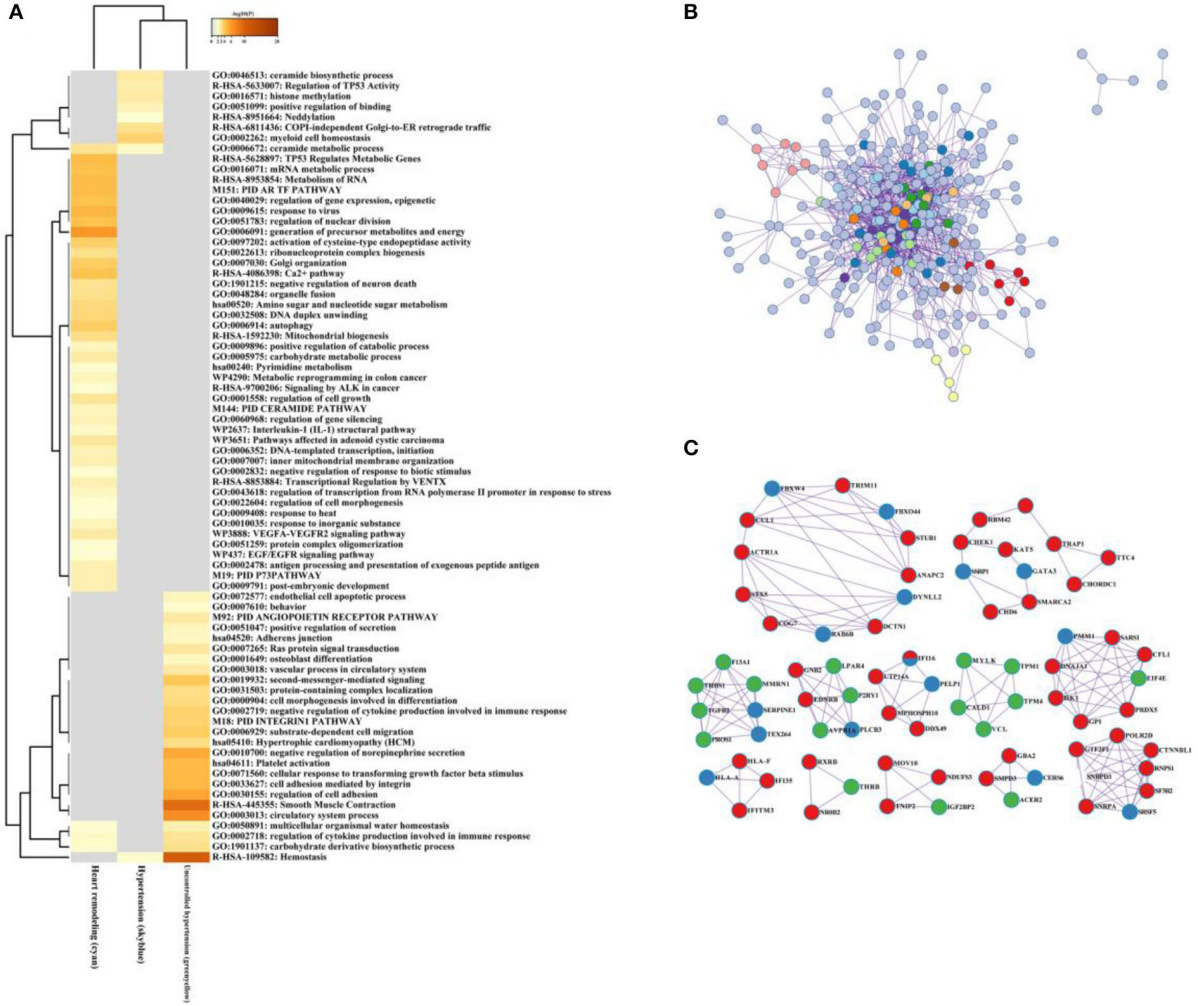
The multiple gene module analysis across hypertension, uncontrolled hypertension, and hypertension with left ventricular remodeling. **(A)** Functional enrichment analysis for the multiple gene module. **(B,C)** Network construction, and hub genes selection and interaction for multiple gene module across hypertension, uncontrolled hypertension, and hypertension with left ventricular remodeling.

As illustrated in Figure 8A, the ceramide biosynthetic process, regulation of TP53 activity, histone methylation, and COPI-independence Golgi-to-ER retrograde traffic were enriched in the hypertension group. The generation of precursor metabolites and energy, Golgi-organization, and Ca^2+^ pathway were involved in hypertension in the left ventricular remodeling group, while negative regulation of norepinephrine secretion, smooth muscle contraction, and hemostasis significantly enriched in the uncontrolled hypertension group.

After the protein–protein interaction (PPI) network construction and hub gene establishment, Figures 8B,C showed that there are different intrinsic gene sets and interaction–interaction across general hypertension, hypertension with left ventricular remodeling, and uncontrolled hypertension. The full list of genes is shown in Supplementary Table 5.

## Discussion

The difficulty in the treatment of hypertension lies in the high incidence and multiple organ involvement. In genetics, the polymorphism of gene loci and the heterogeneity of the population have become obstacles to the gene therapy of hypertension (27, 28). Despite many antihypertensive drugs available, an expressive number of patients continue with uncontrolled hypertension, which was defined as a mean systolic and/or diastolic BP ≥140/90 mmHg based on the Seventh Joint National Committee (JNCVII) on Detection, Evaluation, and Treatment of High Blood Pressure.

In this study, we have comprehensively investigated the transcriptome characteristics related to hypertension from three perspectives, such as general hypertension, hypertension with left ventricular remodeling, and uncontrolled hypertension. The finding that immune/inflammatory response pathways were significantly overrepresented in hypertension from GSE24752 was consistent with the study of Korkor et al. (14) and Wei et al. (23). Interestingly, we found that the biological processes related to blood metabolism, such as “heme metabolism” and “coagulation” were also significantly enriched in the hypertension group (Figures 4A,D).

The advantages associated with our study are as follows: (1) This is the first data-mining study to systematically identify peripheral blood transcriptome characteristics of hypertension at different stages, such as hypertension, hypertension with left ventricular remodeling, and uncontrolled hypertension by means of bioinformatics methods. (2) Considering the expression profile from peripheral blood, blood samples have great advantages in early clinical disease diagnosis compared with tissue samples. (3) Our study suggested that FBXW4 and other 13 genes were unique to the hypertension group, TRIM11 and other 40 genes were mainly involved in hypertension with left ventricular remodeling group, while the other 18 genes including F13A1 significantly enriched in the uncontrolled hypertension group. However, our study has its own limitations. (A) More reliable results would be obtained from a larger sample size; (B) our finding was not verified by experiments, mainly by the bioinformatics method; (C) the level of gene expression in peripheral blood is low, and differential expression analysis may not be applicable; and (D) this may not be common sense that we have no detectable DEGs from hypertension with left ventricular remodeling and uncontrolled hypertension vs. control group. Because our study is a pure bioinformatics analysis based on the GEO database, further biological experiments are needed to validate our results.

In summary, our study showed that there are many gene changes in different stages of hypertension (Figure 8C). Moreover, the dynamic changes in immune metabolism and inflammation are the most significant biological changes in hypertension. Gene microarray based on peripheral blood has the potential to screen the target molecules with diagnostic and therapeutic potential for heart failure.

## Web source and software

Gene Expression Omnibus (GEO): https://www.ncbi.nlm.nih.gov/geo/

Limma: http://bioconductor.org/packages/release/bioc/html/limma.html

GEOquery: http://bioconductor.org/packages/release/bioc/html/GEOquery.html

GSE24752 dataset: https://www.ncbi.nlm.nih.gov/gds/?term=GSE24752

GSE75360 dataset: https://www.ncbi.nlm.nih.gov/gds/?term=GSE75360

GSE74144 dataset: https://www.ncbi.nlm.nih.gov/gds/?term=GSE74144

GSE71994 dataset: https://www.ncbi.nlm.nih.gov/gds/?term=GSE71994

RVA: https://github.com/cran/RVA

WGCNA: https://horvath.genetics.ucla.edu/html/CoexpressionNetwork/Rpackages/WGCNA/

Metascape: https://metascape.org/gp/index.html

Cytoscape: https://cytoscape.org/.

## Data availability statement

The datasets presented in this study can be found in online repositories. The names of the repository/repositories and accession number(s) can be found in the article/ Supplementary material.

## Author contributions

C-yY and X-wZ designed the study and performed the bioinformatics analysis. YG drafted the manuscript. Y-cJ was in charge of language editing. All authors read and approved the final manuscript.

## Funding

This research was supported by the Huaian City Science and Technology Project (HAB202025).

## Conflict of interest

The authors declare that the research was conducted in the absence of any commercial or financial relationships that could be construed as a potential conflict of interest.

## Publisher's note

All claims expressed in this article are solely those of the authors and do not necessarily represent those of their affiliated organizations, or those of the publisher, the editors and the reviewers. Any product that may be evaluated in this article, or claim that may be made by its manufacturer, is not guaranteed or endorsed by the publisher.

## Abbreviations

HT: hypertension
HT-LVR: hypertension with left ventricular remodeling
UN-HT: uncontrolled hypertension
DEGs: differentially expressed genes
GO: Gene Ontology
KEGG: Kyoto Encyclopedia of Genes and Genomes
BP: biological processes
CC: cellular components
MF: molecular functions
PPI: protein-protein interaction
GSEA: Gene Set Enrichment Analysis
WGCNA: weighted gene co-expression network analysis.
